# Supplementary material for: Female community health volunteers’ knowledge and confidence in providing community-based diabetes self-management support in Nepal: A biphasic mixed method evaluation
Source: PLOS Glob Public Health. 2026 Mar 12;6(3):e0006089. doi: 10.1371/journal.pgph.0006089 (PMC12981428; doi:10.1371/journal.pgph.0006089)
Supplement: S1 Text — (DOCX) [file pgph.0006089.s001.docx]

# QUESTIONNAIRE FOR PRE-TEST AND POST-TEST

# ENGLISH QUESTIONNAIRE

## Basic Demographic Information

A. Please, fill in the following information

1. Age:
2. Sex:
3. Educational Status

a. Literate

b. Illiterate

1. If, literate highest level of formal education completed:
2. Do you have any other occupation other than being an FCHV and your housework?
   1. Yes  ☐
   2. No  ☐
3. If yes, please describe it:

**Pre-test questionnaire**

1. Have you heard of diabetes?
2. Yes ☐
3. No ☐
4. Diabetes occur either from lack of insulin or when our body doesn’t respond well to insulin.
5. True  ☐
6. False  ☐
7. Don’t know ☐
8. Diabetes can cause:
9. Heart attacks ☐
10. Heart failure ☐
11. Stroke ☐
12. Kidney damage ☐
13. All of the above  ☐
14. Diabetes type 2 (the type where body doesn’t respond well to insulin) occurs from unhealthy diet, inadequate exercise and obesity. It can be cured.
15. True  ☐
16. False  ☐
17. Don’t know ☐
18. Frequent urination and thirst are signs of diabetes.
19. True  ☐
20. False  ☐
21. Don’t know ☐
22. Do FCHV have a role in diabetes management?
23. True  ☐
24. False  ☐
25. Do you routinely counsel people with diabetes to modify their lifestyle?
26. Yes ☐
27. No ☐
28. What are the factors that may hinder Female Community Health Volunteers (FCHVs) from effectively engaging in activities related to diabetes prevention and control within their community? (Multiple choices) (B)

Lack of Training and orientation ☐

Multiple engagement of FCHVs in different health programs ☐

Lack of incentives and motivation ☐

Lack of Community belief and attitude towards FCHVs skills ☐

1. What is your skill level in counseling people to modify their lifestyle to manage diabetes?
2. I don’t have any skills ☐
3. I only know very little ☐
4. I know some, but it is not enough to counsel ☐
5. I can counsel, but only with some assistance ☐
6. I can independently counsel ☐

**Post test questionnaire**

1. Diabetes occur either from lack of insulin or when our body doesn’t respond well to insulin.

1. True  ☐
2. False  ☐
3. Don’t know ☐

2. Diabetes can cause:

1. Heart attacks ☐
2. Heart failure ☐
3. Stroke ☐
4. Kidney damage ☐
5. All of the above  ☐

3. Diabetes type 2 (the type where body doesn’t respond well to insulin) occurs from unhealthy diet, inadequate exercise and obesity. It can be cured.

1. True  ☐
2. False  ☐
3. Don’t know ☐

4. Frequent urination and thirst are signs of diabetes.

1. True  ☐
2. False  ☐
3. Don’t know ☐

5. Do FCHV have a role in diabetes management?

1. True  ☐
2. False  ☐
3. Don’t know ☐

6. What is your skill level in counseling people to modify their lifestyle to manage diabetes?

1. I don’t have any skills ☐
2. I only know very little ☐
3. I know some, but it is not enough to counsel ☐
4. I can counsel, but only with some assistance ☐
5. I can independently counsel ☐
