# Supplementary material for: Female community health volunteers’ knowledge and confidence in providing community-based diabetes self-management support in Nepal: A biphasic mixed method evaluation
Source: PLOS Glob Public Health. 2026 Mar 12;6(3):e0006089. doi: 10.1371/journal.pgph.0006089 (PMC12981428; doi:10.1371/journal.pgph.0006089)
Supplement: S2 Text — (DOCX) [file pgph.0006089.s002.docx]

**QUESTIONNAIRE FOR FGD**

**Phase 1**

| **Theme** | **Questions** |
| --- | --- |
| **Diabetes related questions** | |
| Knowledge | 1. Please describe what you know about diabetes. |
| Efficacy | 1. Please describe your confidence and skills in counseling people to change lifestyle to reduce diabetes risk. |
| Barriers | 1. What does it take for you to confidently counsel people to change lifestyle to reduce diabetes risk? Please describe what barriers you have. |

**Phase 2**

| **Theme** | **Questions** |
| --- | --- |
| **Diabetes related questions** | |
| Knowledge | 1. Please describe what you know about diabetes. |
| Efficacy | 1. When compared with 10 months ago, please describe your confidence in counseling people to change lifestyle to reduce diabetes risk. |
| Barriers | 1. What barriers and facilitators did you face when counseling people about diabetes risk in the past 10 months? |

*FCHVs role on the prevention and control of NCDs in community:*

1. What were the role of FCHV in the community after training?

2. How many times did you offer the intervention for each of these diseases?

3. In your opinion, what is the level of acceptance of your intervention in the community? (education/information delivered by the FCHVs among the community after the training)

4. Will you be able to participate in such trainings regarding NCDs and conditions in the future?

5. Would you like to share anything about your experience regarding this training?

**Nepali Questionnaire:**

**Phase 1**

| **विषयवस्तु** | **प्रश्नहरु.** |
| --- | --- |
| **मधुमेह (४ प्रश्नहरू)** | |
| मधुमेह बारेमा ज्ञान र धारण | १. कृपया तपाईलाई मधुमेहको बारेमा के थाहा छ वर्णन गर्नुहोस्? |
| आत्म-प्रभावकारिता | १. मधुमेह को जोखिम कम गर्न को लागी प्रदान गरिने सुझाब अथवा परामर्स दिन आफुमा अहिले भएको सिप र आत्मबिस्वासको बर्णन गर्नु होस? |
| बाधा | १ मधुमेहको जोखिम कम गर्न जीवनशैली परिवर्तन गर्न मानिसहरूलाई आत्मविश्वासका साथ परामर्श दिन तपाईंलाई के चाहिन्छ ? साथै तपाईंले काम गर्दा कस्ता खालका बाधाहरु आइपर्छन जस्तो लाग्छ? |

**Phase 2**

| **विषयवस्तु** | **प्रश्नहरु.** |
| --- | --- |
| **मधुमेह (४ प्रश्नहरू)** | |
| मधुमेह बारेमा ज्ञान र धारण | १. कृपया तपाईलाई मधुमेहको बारेमा के थाहा छ वर्णन गर्नुहोस्? |
| आत्म-प्रभावकारिता | १. गत १० महिनाको तुलनामा, मधुमेह को जोखिम कम गर्न को लागी प्रदान गरिने सुझाब अथवा परामर्स दिन आफुमा अहिले भएको सिप र आत्मबिस्वासको बर्णन गर्नु होस? |
| बाधा | १ . विगत १० महिनामा, , मधुमेह को जोखिम कम गर्ने सम्बन्धि परामर्श प्रदान गर्दा तपाइँले समुदायमा कस्ता बाधा र सहजकर्ताहरुको सामना गर्नुभयो? |

**समुदायमा नसर्ने रोगको रोकथाम र नियन्त्रणमा महिला स्वास्थ्य सोयम सेविकाको भूमिका:**

1. नसर्ने रोग सम्बन्धी तालिम प्राप्त गरिसकेपछि समुदायमा महिला स्वास्थ्य स्वयंसेविकाको भूमिका  कस्तो रह्यो ?

2. यी माथि उल्लेखित सबै रोगहरु मध्ये कतिपटक प्रत्येक रोगको लागि समुदायमा कार्यहरु गर्नुभयो?

3. तपाईंको विचारमा तपाईंहरुले   प्रदान गरेको  नसर्ने रोग सम्बन्धी  कार्यहरुमा समुदायको मानिसको  स्वीकृति  स्तर कस्तो रह्यो ? (जस्तै तपाईंहरुले दिनुभएको  स्वास्थ्य सम्बन्धि जानकारीहरू)

4. के तपाईहरु भविष्यमा अब यस्तै नसर्ने रोग सम्बन्धी तालिम भयो भने फेरिपनि सहभागी हुन सक्नुहुन्छ?

5. तपाईहरु यो training सँग सम्बन्धित केही आफ्ना अनुभवहरु हामी माझ  सुनाउन चाहनुहुन्छ?
