# Supplementary material for: Female community health volunteers’ knowledge and confidence in providing community-based diabetes self-management support in Nepal: A biphasic mixed method evaluation
Source: PLOS Glob Public Health. 2026 Mar 12;6(3):e0006089. doi: 10.1371/journal.pgph.0006089 (PMC12981428; doi:10.1371/journal.pgph.0006089)
Supplement: S4 Text — (DOCX) [file pgph.0006089.s004.docx]

**Theme 1: Knowledge about diabetes**

Phase 1

Quote 1: त्यति धेरै थाहा छैन। गुलियो नै खाएर मधुमेह लाग्ने भन्ने हुदैन। (Ward No. 7, Phase 1)

Quote 1: “I don’t know much, but I do know that eating sweets isn’t the only cause of diabetes.” – (Ward No. 7, Phase 1)

Phase 2:

Quote 2: मधुमेह भनेको एक प्रकारको नंसर्ने रोग हो । सुगर हुने त्योचाहिँ नसर्ने रोगको हेल्थ फाउन्डेसनले सिकाएको। मधुमेह रोग शरीरमा गुलियोको मात्रा धेरै भएर हुन्छ। यसका लक्षणहरु दुब्लाउँदै जाने, पानी धेरै तिर्खा लाग्ने, मुख सुक्ने, हात हात खुट्टा झमझमाउने हुन्छ। (Ward No. 6 Phase 2)

Quote 2: “Diabetes is a type of non-communicable disease that leads to increased blood sugar levels. We learned this from the Health Foundation. It occurs when the sugar level in the body becomes too high. Its symptoms include unexplained weight loss, excessive thirst, dry mouth, and a tingling sensation in the hands and feet.” - (Ward No. 6 Phase 2)

**Theme 2: Diet and lifestyle counseling**

Phase 1

Quote 3: गुलियो कुरा खानु हुदैन भन्ने थाहा छ, त्यति हो। मैले पहिला तेस्तो केहि सलाहा सुझाव देको छैन। ( Ward No.3 Phase 1)

Quote 3: “I only know that people with diabetes shouldn’t eat sweet things. I haven’t given any advice or suggestions before.” ( Ward No.3 Phase 1)

Phase 2

Quote 4: “सुगर भएको मान्छेलाई साथै भन्यो भने खाना एकदम टाइममा खानु पर्छ , टाइममा बिहान २ ओटा रोटी र तरकारी खाने, भात नखाने गुलियो कुरा खान भएन ,सकिन्छ रोटी खाने व्यायम गर्ने। नभए पछि गएर यसले दुख दिन्छ भनेर भन्न सकिन्छ। धेरैलाई छ धेरैले थाहा पाइसकेको छन् अहिले।" ( Ward No.3, Phase 2)

Quote 4: “Those who have diabetes should eat their meals on time. In the morning, they should have two flatbreads with vegetables timely. They should avoid rice and sweet foods. If possible, they should stick to flatbread and make sure to exercise. Otherwise, it can lead to problems later on. I share this advice with them. These days, many people have diabetes and are already aware of some of these things.” (Ward No.3, Phase 2)

**Theme 3: FCHVs' Confidence**

Phase 1:

Quote 5: हामी यसको बारेमा पढेको त छैनौ तर अलि अलि थाहा भाको कुरा हामीले भन्छौ। (Ward No.3 Phase 1)

Quote 5: “We haven’t read about this but we share whatever little we do know with others.” (Ward No.3 Phase 1)

Phase 2:

Quote 6: पहिला बुझेको थियेनम, तालिम पाए पछि त मज्जाले भन्न सक्छम हामीले। सुगर मदुमेह भनेको यस्तो रोग हो यो चेक गराउनु पर्छ, खान पनि अलिकति कन्ट्रोल गरेर खानु पर्छ भनेर भन्न सक्छम। (Ward No. 4 Phase 2)

Quote 6: “Previously, we didn’t understand much. But after receiving training, we can now counsel others more easily(confidently). We explain that diabetes is a condition that requires checkups and proper control of food intake. We’re now able to share this information.” (Ward No. 4 Phase 2)

Quote 7: मलाई नै सुगर भएको कारणले गर्दा त्यस्तो बाधाहरु आएनन्, आफूलाई नै भएको कारणले गर्दा कस्तो कस्तो लक्षणहरु हुन्छन्, तपाईलाई कस्तो भएको थियो मलाई अलिकति बढी भोक लाग्छ निद्रा लाग्दैन टाउको एकदमै झम्म गर्छ भन्नुहुन्छ सल्लाह लिन आउनुहुन्छ,आउने बेला मधुमेहको बारेमा हातखुट्टा झमझमाउने, मुख सु सुक्खा हुने , नाकमुख चकरक्क सुक्ने हुन्छ त्यही पनि एकपटक जानुहोस् चेक गराउनुहोस् भनेर सल्लाह दिइन्छ। (Ward No. 6, Phase 2)

Quote 7: “I have diabetes myself, so I didn’t face many barriers. Since I experience it firsthand, I know many of the symptoms. I often ask others what symptoms they are having. They tell me they feel hungrier, can’t sleep well, have headaches, and they come to me for advice. When they do, I explain what diabetes is. I tell them they might feel tingling in their hands and feet, or have a dry mouth and dry nose. So, I counsel them to go get a checkup.” (Ward No. 6, Phase 2)

**Theme 4: Training and education**

Phase 1

Quote 8: संग संस्था आउछन, एक चोटी तालिम दिन्छन, फेरी का जान्छ जान्छ। त्यसैले हामी अपडेट हुदैनौ। (Ward No. 6, Phase 1)

Quote 8: Some organizations come and provide training just once, then never return. As a result, we aren’t updated. (Ward No. 6, Phase 1)

Quote 9: तालिम दिएसी सक्छम हुन्छौ। हामीलाइ जानकारी नै छैन भने हामीले अरुलाई कसरि सल्लाह सुझाव दिन सक्छौ। (Ward No. 3, Phase 1)

Quote 9: We can become capable if we receive proper training. Without knowledge, how can we offer suggestions and advice to others? (Ward No. 3, Phase 1)

Phase 2:

Quote 10: हामी पुरै तालिम प्राप्त भए पछि समुदायमा फैलाउने कुरा हो, आफु पहिले बिस्वस्त हुन पर्यो, म सक्छु त्यहा गएर काम गर्न भन्ने। (Ward No. 3, Phase 2)

Quote 10: We can only spread knowledge in the community if we receive complete training. First, we need to feel fully confident and say to ourselves, “Yes, I can go and work in the community.” (Ward No. 3, Phase 2)

**Theme 5: Barriers**

**Subtheme 1: Workload**

Phase 1

Quote 11: तालिमको आवस्यक छ। तालिम एक पटक दिएर हुदैन। १ पटक दिएर वर्षौ वर्षौ हामी दिमागमा राख्न सक्दैनौ। हामीलाई तालिम ६-६ महिनामा चाहिन्छ। किन भने हामीलाई धेरै काम हरु हुन्छ, धेरै काममा सम्लंग हुन पर्छ, बिर्सिन्छ। हामी महिला स्यवेम सेविकाले ३५ ओटा काम गर्छम, गर्भवती महिला देखि बुढाबुढिको लागि काम गर्नु पर्छ। (Ward No. 6, Phase 1)

Quote 11: There is a need for regular training. A one-time training is not enough. It’s difficult to retain all the information from a single session over the years. We need training every six months. Since we are involved in so many different tasks, we tend to forget. As Female Community Health Volunteers, we are responsible for around 35 different types of work—ranging from caring for pregnant women to supporting the elderly. ( Ward No. 6, Phase 1)

**Subtheme 2: Incentives**

Phase 1

Quote 12: तालिमसँगसंगै हामीलाई, सोयम्सेविकालाई तलव नदिए पनि, भत्ता त बढाए हुन्थ्यो, जस्तै सधैं त्यो ४०० ले के हुन्छ, कम्तिमा १००० गराइदियो भने हामीलाई पनि काम गर्न उत्साह जग्थ्यो..( Ward No. 3, Phase 1)

Quote 12: “*There should be provision of training along with increased incentives though there is no salary for us. Rs 400 is not sufficient for us, at least there should be provision of Rs. 1000 so [we] will be encouraged to do our responsibility*.” - Ward No. 3, Phase 1

**Subtheme 3: Community’s receptiveness towards counseling**

Phase 1

Quote 13: “केवल सल्लाह दिनुहुन्छ कि अरु के दिनु भयो वा भन्नुहुन्छ गाउँका मानिसले, त्यसो भए औषधि पनि दिन पाएको भए राम्रो हुने थियो.” (Ward No. 6, Phase 1)

Quote 13: “*Community people said that, we only do counseling without providing anything. So, it would be better if there is provision of medicine*.” Ward No. 6, Phase 1

Phase 2

Quote 14: "हजुरले भन्नुभयो हामीले चेक गर्यौं। कसैले ठिकै छ भन्नु हुन्छ, कसैले थोरै बढ्न थालेको रैछ , अलि कन्ट्रोल गर्नुहोस भन्नु भो डाक्टरले भनेर भन्नु हुन्छ। हामीले भनेको कुरा गर्नु हुन्छ , यस्तो किन गरेको भन्नु हुन्न , राम्रो हो राम्रो भन्नु भो भन्नु हुन्छ। .” ( Ward No. 4, Phase 2)

Quote 14: “*Some say that they are doing checkup as per our guidance, some said they are fine, some said their sugar level is increasing and doctor is suggesting having control on diet. They said that the things we were saying was good*.” - Ward No. 4, Phase 2

Quote 15: "हामीले दिएको सल्लाह सुझाब मान्नु हुन्छ, सुन्नु हुन्छ, ठिकै हो भन्नु हुन्छ, चेक गराउनु हुन्छ।" (Ward No. 4, Phase 2)

Quote 15: “*They follow our suggestions and advices, they listen to us, they said we are right and they do checkup*.” - Ward No. 4, Phase 2
