# Supplementary material for: Female community health volunteers’ knowledge and confidence in providing community-based diabetes self-management support in Nepal: A biphasic mixed method evaluation
Source: PLOS Glob Public Health. 2026 Mar 12;6(3):e0006089. doi: 10.1371/journal.pgph.0006089 (PMC12981428; doi:10.1371/journal.pgph.0006089)
Supplement: Attachment — Submitted filename: PGPH-D-25-03684_PM.pdf [file pgph.0006089.s005.pdf]

PLOS Global Public Health

Female community health volunteers’ knowledge and confidence in providing community-based diabetes self-management support in Nepal: A biphasic mixed method evaluation

--Manuscript Draft--

Manuscript Number:	PGPH-D-25-03684
Article Type:	Research Article
Full Title:	Female community health volunteers’ knowledge and confidence in providing community-based diabetes self-management support in Nepal: A biphasic mixed method evaluation
Short Title:	FCHVS' knowledge and confidence in diabetes self-management support in Nepal
Corresponding Author:	Susagya Bhusal, BPH<br>Health Foundation Nepal<br>Kathmandu, NEPAL
Order of Authors:	Ada Thapa
	Susagya Bhusal
	Omkar Jha
	Adipti Pantha
	Sudim Sharma
	Austin Seals
	Madhav KC
	Archana Shrestha
	Bibhav Acharya
	Yashashwi Pokharel
Keywords:	Non-communicable Disease, Diabetes self-management, Female Community Health Volunteers, implementation science, mixed method, community-based training
Abstract:	Self-management support by community health workers, referred to as Female Community Health Volunteers (FCHVs) in Nepal, can enhance diabetes care in resource-constrained settings. We assessed FCHVs’ knowledge, perceived self-efficacy, and barriers to supporting diabetes self-management before and after training. Guided by the Health Belief Model, we conducted a biphasic, Qual + quan, concurrent, embedded mixed-methods evaluations among 28 FCHVs in four wards in rural mid-Western Nepal. We evaluated the program into two phases, each consisting of focus group discussions (FGDs); trainings that included didactics, hands-on-practice, and role play demonstrations; and pre- and post-training surveys. Phase 2 occurred 11 months after Phase 1 (January 5-8, 2021). We conducted trainings and evaluations over 4-5 hours/ward/phase. FGD data were analyzed using both deductive and inductive coding and emerging themes were then examined for patterns and relationships among the codes. We summarized quantitative data using descriptive statistics and integrated with qualitative data during interpretation. FCHVs’ mean age was 48 years, 32.1% had completed secondary education, and 75% had served as FCHVs for >10 years. We identified five themes in qualitative analysis: (1) diabetes knowledge, (2) diet and lifestyle counseling, (3) FCHV’s confidence, (4) training and education, and (5) barriers and motivators for community-based self-management support. Mixed-methods evaluations showed knowledge of diabetes and confidence in self-management skills improved after the training. Although barriers, such as inadequate incentives, persisted in Phase 2, communities’ trust in FCHV as diabetes self-management supporter improved. These findings suggest that FCHVs are willing to take a greater role in diabetes self-management support, and that even short training sessions can enhance their confidence and knowledge. Ensuring appropriate incentives, ongoing trainings and system-level support are important for sustainability

Powered by Editorial Manager® and ProduXion Manager® from Aries Systems Corporation

	of such programs.
**Additional Information:**	
**Question**	**Response**
**Financial Disclosure**<br><br>Enter a financial disclosure statement that describes the sources of funding for the work included in this submission and the role the funder(s) played. This includes grants and any commercial funding of the work or authors.<br><br>This statement will be typeset if the manuscript is accepted for publication.<br><br>*Please review the submission guidelines and the instructions link below for detailed requirements and guidance.*	Dr. Yashashwi Pokharel received funding from the Health Foundation Nepal.
**Competing Interests**<br><br>On behalf of all authors, disclose any competing interests that could be perceived to bias this work.<br><br>This statement will be typeset if the manuscript is accepted for publication.<br><br>*Please review the instructions link below and PLOS Global Public Health's competing interests policy to determine what information must be disclosed at submission.*	Dr. Bibhav Acharya is an academic editor for PLOS GPH.
**Data Availability**<br><br>Before publication, Authors are required to make fully available and without restriction all data underlying their findings. Please see our PLOS Data Policy page for detailed information on this policy.	Data are available upon reasonable request.

A **Data Availability Statement**, detailing where the data can be accessed, is required at first submission. Insert your Data Availability Statement in the box below.<br><br>Please see the data reporting section of our submission guidelines for instructions on what you need to include in your Data Availability Statement.<br><br>This statement will be typeset if the manuscript is accepted for publication.<br><br>*PLOS allows rare exemptions to address legal and ethical concerns. If you have legal or ethical restrictions, please use the box below to detail these in full sentences for the Journal team to consider.*	

Powered by Editorial Manager® and ProduXion Manager® from Aries Systems Corporation

Manuscript

Click here to access/download;Manuscript;Manuscript_Final_26Nov.docx

Full Title: Female community health volunteers' knowledge and confidence in providing community-based diabetes self-management support in Nepal: A biphasic mixed method evaluation

Short Title: FCHVS' knowledge and confidence in diabetes self-management support in Nepal

Ada Thapa[1,2¶], Susagya Bhusal[1¶*], Omkar Jha[1], Adipti Pantha[1], Sudim Sharma[1], Austin Seals[3], Madhav KC[5], Archana Shrestha[6], Bibhav Acharya[7], Yashashwi Pokharel[1,3,4]

[1].Health Foundation Nepal, Dang, Nepal
[2].Center for Integration Science in Global Health Equity, Department of Medicine, Brigham and Women's Hospital, Boston, MA, USA
[3].Departments of Internal and Cardiovascular Medicine, Atrium Health Wake Forest Baptist, Wake Forest University, School of Medicine, Winston-Salem, NC 27101 USA
[4].Department of Implementation Science, Office of Global Health, Atrium Health Wake Forest Baptist, Wake Forest University, School of Medicine, Winston-Salem, NC 27101 USA
[5].Department of Epidemiology, University of Nebraska Medical Center, Omaha, NE 68198 USA
[6].Department of Public Health, Kathmandu University School of Medical Sciences, Dhulikhel, Nepal
[7].University of California, San Francisco, California, USA,

*Corresponding Author:
Email: bhusal.susagya06@gmail.com (SB)

[¶]These authors contributed equally to this work.

Abstract

Self-management support by community health workers, referred to as Female Community Health Volunteers (FCHVs) in Nepal, can enhance diabetes care in resource-constrained settings. We assessed FCHVs' knowledge, perceived self-efficacy, and barriers to supporting diabetes self-management before and after training. Guided by the Health Belief Model, we conducted a biphasic, Qual + quan, concurrent, embedded mixed-methods evaluations among 28 FCHVs in four wards in rural mid-Western Nepal. We evaluated the program into two phases, each consisting of focus group discussions (FGDs); trainings that included didactics, hands-on-practice, and role play demonstrations; and pre- and post-training surveys. Phase 2 occurred 11 months after Phase 1 (January 5-8, 2021). We conducted trainings and evaluations over 4-5 hours/ward/phase. FGD data were analyzed using both deductive and inductive coding and emerging themes were then examined for patterns and relationships among the codes. We summarized quantitative data using descriptive statistics and integrated with qualitative data during interpretation. FCHVs' mean age was 48 years, 32.1% had completed secondary education, and 75% had served as FCHVs for >10 years. We identified five themes in qualitative analysis: (1) diabetes knowledge, (2) diet and lifestyle counseling, (3) FCHV's confidence, (4) training and education, and (5) barriers and motivators for community-based self-management support. Mixed-methods evaluations showed knowledge of diabetes and confidence in self-management skills improved after the training. Although barriers, such as inadequate incentives, persisted in Phase 2, communities' trust in FCHV as diabetes self-management supporter improved. These findings suggest that FCHVs are willing to take a greater role in diabetes self-management support, and that even short training sessions can enhance their confidence and

knowledge. Ensuring appropriate incentives, ongoing trainings and system-level support are important for sustainability of such programs.

Introduction

Non communicable diseases (NCDs) such as heart disease, stroke, and diabetes are the leading causes of mortality and morbidity globally [1]. Approximately 537 million adults worldwide are currently living with diabetes [2]. This number is projected to increase by 46% by 2045, with an estimated 1 in 8 adults having diabetes. About 80% of affected individuals reside in low- and middle-income countries (LMIC) with limited resources [1, 2]. Managing diabetes in these settings presents numerous challenges, including limited healthcare professionals, medications, and challenges in improving patients' self-management skills. Given these challenges, community-based, sustainable and scalable diabetes care approaches that do not solely rely on traditional healthcare providers can augment diabetes care [3, 4].

Improving diabetes self-management skills within communities is an important but neglected aspect of diabetes care. Self-management skills enhance knowledge about diabetes, medication adherence, adoption of healthy lifestyle modifications and confidence in chronic disease management [5, 6]. Community-based diabetes self-management support by community health workers (CHWs) therefore is highly relevant in low-resource settings [3, 4]. CHWs serve as a vital link between healthcare systems and communities, facilitating services and providing culturally appropriate care [7]. By leveraging local knowledge and trust, CHW programs effectively provide contextually appropriate services within communities. Nevertheless, aligning the fitness of CHW programs to local context, including CHW's needs, is important, particularly when CHWs are involved in new areas of healthcare like diabetes. Female Community Health Volunteers (FCHVs) are the most common types of CHW in Nepal and have been an integral

part of the healthcare system since 1988. There are ~50,000 FCHVs in Nepal chosen by local women-led civic group called the Mother's group [8, 9]. FCHVs typically receive basic training from the Ministry of Health and Population focusing on promoting healthy behaviors among communities, safe motherhood, child health, family planning, and distributing contraceptives, oral rehydration solutions packets, vitamin A capsules, iron tablets, treating pneumonia, referring severe cases to health institutions, and providing motivation and education [3, 8-11]. Recently there has been growing interest in expanding their involvement to non-communicable diseases (NCDs), including diabetes self-management support [7, 12-15]. However, before expanding such program, evaluating whether FCHVs possess the necessary knowledge and confidence in delivering diabetes care is essential. An assessment of their skills, training needs, potential barriers, and ability to retain information is important to understand the program's success. This is particularly relevant given the considerable variability in FCHV's education and background [12, 14]. Therefore, we conducted a biphasic concurrent mixed-methods evaluation of FCHVs' knowledge, perceived self-efficacy, and barriers in providing diabetes self-management support 11 months after an initial training (Figure 1).

Figure 1. Study Procedure

Materials and Methods

Setting and Sampling

Health Foundation Nepal (HFN), a non-profit organization, has been carrying out a community-based prospective epidemiological and implementation program, NCD Nepal study, in four wards of Dang District, located Southwest of Kathmandu, the capital [16]. The NCD Nepal study is an ongoing longitudinal study where FCHVs project is embedded. The NCD Nepal study

collaborates with FCHVs to coordinate program activities like connecting with the study participants for follow up [16]. Prior to this study, the FCHVs were not trained on diabetes self-management.

We obtained contact information and called all 45 FCHVs from the four wards of the NCD Nepal study area informing about the current study. Due to COVID-19, gathering was restricted to seven people according to local rules. We could not engage FCHVs who were unreachable by phone. Three FCHVs declined to participate due to other commitments. We conducted a second round of calls with FCHVs who had initially agreed to participate. Once seven FCHVs from each ward confirmed participation, we did not contact the remaining FCHVs to comply with the gathering policy. We invited FCHVs to local schools and health centers for training and evaluated their readiness in using community-based self-management support for diabetes, hypertension, hyperlipidemia and tobacco use. The current study is focused only on diabetes. We obtained written informed consent from all FCHVs. All 28 FCHVs completed the study. In accordance with local norms, we provided each FCHV ~3 USD per phase and a light snack and tote bag containing educational pamphlets about chronic diseases.

Data Collection

We collected both qualitative and quantitative data simultaneously but independently across two phases (Figures 1-2). The phase 1 took place from January 5, 2021, to January 8, 2021, and phase 2 took place from October 9, 2021 to October 2021. Each phase included a FGD, a training session, and two diabetes-focused survey questions before and after each training in every ward (Figure 1). We completed all activities in each phase within 4-5-hours.

Fig 1. Study Timeline

Fig 2. Flowchart of Mixed Method Design

Incorporating constructs from the Health Belief Model (HBM), specifically knowledge, self-efficacy, and barriers, we designed interview guides and surveys to evaluate FCHVs' understanding of diabetes, their confidence in counseling, and perceived obstacles to providing community-based diabetes self-management support [17, 18]. The HBM proposes six socio-behavioral constructs influencing health behaviors. We selected three constructs – knowledge, self-efficacy, and barriers - to understand how FCHVs perceive and provide self-management support reflecting internal and external factors that influence their practices. We piloted the interview guides and surveys among lay persons and study staff to make some minor changes for clarity and flow.

Phase 1

Focus Group Discussion (FGD): We conducted the FGDs in the Nepali language within local schools and health centers maintaining privacy. We audio-recorded the interviews that lasted between 45-90 minutes.

S*urvey 1:* At baseline, we collected sociodemographic information and concurrently administered a 9-item survey (S1) about diabetes concurrently with FGDs (S2).

Training 1: The primary objective of the training was to enhance FCHVs' knowledge and skills, empowering them to provide effective diabetes self-management support. The training program included diabetes-focused didactics, hands-on training exercises (such as glucose measurement), and role-play demonstrations. During the hands-on training, participants were educated on normal, borderline, and high glucose levels. A study physician delivered didactics on diabetes management, while project staff facilitated role-play scenarios simulating physician-patient

interactions for diabetes management. The training emphasized diabetes risk factors, sign and symptoms, complications and prevention and control measures, importance and approaches for control, including medications and lifestyle modifications, medication adherence, importance of clinical follow up and community-based care approaches. We used educational materials such as presentations, posters, and pamphlets throughout the session.

Survey 2: Following the training, we administered a second survey, similar to survey 1, consisting of six items to evaluate immediate changes in FCHVs' knowledge after the training (S1). Survey 2 was nearly identical to Survey 1. Items no longer relevant at Survey 2 were excluded.

Phase 2:

Focus group discussion: Eleven months after phase 1, we started phase 2 with FGD like phase 1. We further evaluated barriers and facilitators that FCHVs encountered while providing diabetes-related care in the community. Because FCHVs had not yet engaged in such activities before, this topic was not included in Phase 1 (S2)

*Survey 3***:** We administered a survey comprised of nine items about diabetes concurrently with FGD (S1).

*Training***:** We conducted a refresher training replica of phase 1 that included diabetes-focused didactics, hands-on training exercises (such as checking glucose levels), and role-play demonstrations.

Survey 4: We conducted **s**urvey 4 following the refresher training like survey 3.

Data Analysis

Quantitative analysis

We used R version 4.0.2 for descriptive statistics and Fisher's exact test to detect differences across the phases. A two-sided p-value <0.05 was considered statistically significant. We did not adjust for multiple comparisons. To evaluate a relatively long-term changes in readiness for diabetes self-management support from our training, we compared the results from surveys 1 to 4.

Qualitative analysis

We applied both deductive and inductive coding techniques for qualitative analysis of interview data [19]. Deductive codes were based on pre-established constructs from the HBM, while inductive coding facilitated the emergence of new themes from the data. We derived the themes by identifying and interpreting key patterns and relationships among the codes.

AT and SS reviewed the audio-recorded and transcribed interviews to ensure quality and developed a codebook that was shared with the larger investigative team for finalization. AT then independently applied the finalized codes using NVivo 1221. We developed code summaries to identify emergent themes, which were consolidated by both AT and SS. These themes were then discussed with the broader team to ensure consensus and further refinement of the findings. We aligned the emerging themes along the three HBM constructs (S3 and S4).

Mixed-methods integration

We employed concurrent mixed-method approach, embedding quantitative data with qualitative dominant data (Qual + quan), collecting and analyzing qualitative and quantitative data separately but simultaneously in two phases [20]. We integrated the data together during results interpretation for convergence and complementarity (Figure2) [20].

Ethical Considerations

The current study is the part of ongoing NCD Nepal study[16]. We obtained ethical approval from Nepal Health Research Council (Ref: 2483) and a written consent from each FCHV.

Results

Sociodemographic characteristics

FCHVs' mean age (SD) was 48 (6.8) years. One FCHV was not able to read and write.

Table 1: Sociodemographic characteristics of participating FCHVs (N=28)

**Variables**	**Category**	**N (%)**
Age (years)	Mean= 48, SD= ± 6.79, Min= 30, Max= 59	
	≥40	6)21.4(
	>40	22)79.6)
Educational status	Literate	27)96.4(
	Illiterate	1)3.6(
Highest education completed	Informal Education	2)7.1(
	Primary Education (Grade 1-5)	17)60.7(
	Secondary Education (Grade 6-8)	9)32.1(
Other Occupation	Yes	2)7.1(
)Besides FCHV(	No	26)92.9(
Years of engagement as FCHVs	≥10 years	7)25(
	>10 years	21)75(

FCHV = Female Community Health Volunteer, N= group size, SD= standard deviation

Main Results

We compiled qualitative and quantitative results under five themes: diabetes knowledge; diet and lifestyle counseling; FCHVs' confidence; training and education; and barriers and motivators in community-based counseling delivery (Table 2).

Table 2: Quantitative evaluation of FCHVs' responses

**Themes**	**Phase 1**			**Phase 2**		
	**Survey (S) 1**	**Survey (S) 2**	**p- value**	**Survey (S) 3**	**Survey (S) 4**	**p-value**
**Theme 1: Diabetes Knowledge *(N [%] selecting "True" response)***						
Heard about diabetes	26 (92.9)	NA		28 (0)	NA	
Diabetes occur either from lack of insulin or when our body doesn't respond well to insulin	12 (42.9)	27(96.4)	<0.001	18 (64.3)	27 (96.4)	0.005
Diabetes can cause:						
Heart attack	6 (21.4)	23 (82.1)	<0.001	8 (28.6)	7 (25.0)	1
Heart failure	4 (14.3)	20 (71.4)	<0.001	3 (10.7)	2 (7.1)	1
Stroke	2 (7.1)	18 (64.3)	<0.001	2 (7.1)	0 (0.0)	0.491
Damage kidney	5 (17.9)	17 (60.7)	0.002	4 (14.3)	3 (10.7)	1
Diabetes causes all of the above	7 (25.0)	17 (60.7)	0.014	12 (42.9)	20 (71.4)	0.058
Diabetes type 2 occurs from unhealthy diet, inadequate exercise and obesity. It can be cured.	16 (57.1)	28 (100.0)	<0.001	19 (67.9)	26 (92.9)	0.040
Frequent urination and thirst are signs of diabetes	24 (85.7)	28 (100.0)	0.001	25 (89.3)	28 (100.0)	0.236
**Theme 2: FCHVs confidence *(N [%] selecting "True" response)***						
FCHV has a role in diabetes management	20 (71.4)	28 (100.0)	0.004	24 (85.7)	24 (85.7)	0.1118
**Theme 3: Diet and Lifestyle counseling**						

Skill level in counseling people to modify their lifestyle *(N [%] selecting "I can independently counsel "or "I can counsel, but only with some assistance" or both.*	15 (53.6)	24 (85.7)	0.019	20 (71.4)	27 (96.4)	0.025
Routinely counsel people with diabetes to modify their lifestyle *(N [%] selecting "Yes" response)*	24 (85.7)	NA		12 (42.9)	NA	
**Theme 4: Training and education as a barrier *(N [%] selecting "True" response)***						
Lack of training and education	24 (71.4)	NA		12 (42.9)	NA	
**Theme 5: Barriers and motivators for community-based self-management support *(N [%] selecting "True" response)***						
Community belief and attitude towards FCHVs skills	9 (32.1)	NA		12 (42.9)	NA	
Multiple engagement of FCHVs in different health programs	9 (32.1)	NA		11 (39.3)	NA	
Incentive and motivation	7(25.0)	NA		17 (60.7)	NA	

NA- not applicable as question was not asked

Theme 1: Diabetes Knowledge

FCHVs resoundingly expressed having a role in diabetes care. In Phase 1, quantitative data revealed FCHVs unfamiliarity with the concept of diabetes, which noticeably changed in phase 2 where they were able to identify that type 2 diabetes can be caused by an unhealthy diet, inadequate exercise, and obesity (Table 2). Additionally, most FCHVs in phase 2 understood that diabetes can result from a lack of insulin or poor insulin response. In phase 1, there was less awareness of diabetes-related complications acknowledging that diabetes can cause heart attacks, heart failure, strokes, and kidney damage that improved significantly on Phase 2, although gap still persisted.

Qualitative findings from Phase 1 and Phase 2 illustrated a noticeable change in FCHVs' understanding of diabetes. In Phase 1, FCHVs generally expressed basic awareness of diabetes and its management. While they had heard of diabetes, their responses indicated minimal understanding of its underlying causes, clinical manifestations, and potential health outcomes. As one participant cautiously shared,

> Quote 1: "*I don't know much, but I do know that eating sweets isn't the only cause of diabetes."* – (Ward No. 7, Phase 1)

This response hinted that although FCHVs did not have a comprehensive understanding, they knew that diabetes is multifactorial.

By Phase 2, however, participants expressed a more nuanced and accurate understanding of the condition. FCHVs were not only able to define diabetes but also describe its symptoms and causes with notable clarity and confidence. One participant articulated,

> Quote 2: "*Diabetes is a type of non-communicable disease that leads to increased blood sugar levels. We learned this from the Health Foundation. It occurs when the sugar level in the body becomes too high. Its symptoms include unexplained weight loss, excessive thirst, dry mouth, and a tingling sensation in the hands and feet."* – (Ward No. 6, Phase 2)

While integrating qualitative and quantitative data, it became evident that FCHVs showed progress in their understanding of diabetes, moving from general to more specific expression, suggesting that the training sessions were effective.

Theme 2: Diet and Lifestyle Counseling

From the quantitative findings in Phase 1, 15 FCHVs reported that they could either independently counsel individuals about diabetes or do so with some assistance. By phase 2, the number has increased to 27, suggesting a notable improvement in confidence and skills in providing lifestyle and diabetes related counseling.

In qualitative analysis, we noted that FCHVs at phase 1 had limited skills and confidence in diet and lifestyle counseling. They reported that their understanding rarely extended beyond warning against sugar consumption. They acknowledged a lack of deeper knowledge about dietary or lifestyle modifications as one participant articulated:

> Quote 3: "*I only know that people with diabetes shouldn't eat sweet things. I haven't given any advice or suggestions before*." (Ward No. 3, Phase 1)

In Phase 2 FCHVs were more aware of the broader impact of diet and lifestyle counseling on diabetes management. They understood the importance of self-management counseling and actively incorporated knowledge from the training into counseling community members about the significance of diet and exercise in managing blood sugar levels. One participant gave a detailed description of tailored dietary routine and regular exercise to maintain a healthy lifestyle, demonstrating a comprehensive understanding of diabetes management.

> Quote 4: "*Those who have diabetes should eat their meals on time. In the morning, they should have two flat breads with vegetables on time. They should avoid rice and sweet foods. If possible, they should stick to flatbread and make sure to exercise. Otherwise, it can lead to problems later on. I share this advice with them. These days, many people have diabetes and are already aware of some of these things."* (Ward No. 6, Phase 2)

The integration of quantitative and qualitative findings indicated that knowledge acquisition through structured training and hands on learning experience enhanced FCHVs' counseling practices.

Theme 3: FCHVs' Confidence

FCHVs' confidence in their role to provide diabetes self-management support grew notably after phase 1 training. By phase 2, their confidence stayed high suggesting they retained that sense of ability over time. Qualitative findings showed a notable shift in FCHVs' confidence as community health educators. In Phase 1, FCHVs described a limited but genuine effort to share whatever knowledge they had, despite lacking formal training or comprehensive understanding.

> Quote 5: "*We haven't read about this, but we share whatever little we do know with others*." (Ward No.3, Phase 1)

In phase 2, FCHVs not only reported increased knowledge but also described feeling more capable in delivering health information. One participant noted how the training had equipped them to speak more effectively about diabetes management and counseling strategies.

> Quote 6: "*Previously, we didn't understand much. But after receiving training, we can now counsel others more easily. We explain that diabetes is a condition that requires checkups and proper control of food intake. We're now able to share this information*." (Ward No. 4, Phase 2)

Another participant used her lived experience with diabetes to connect with the community members and provide empathetic, informed support.

> Quote 7: "*I have diabetes myself, so I didn't face many barriers. Since I experienced it firsthand, I know many of the symptoms. I often ask others what symptoms they are having. They tell me they feel hungrier, can't sleep well, have headaches, and they come to me for advice. When they do, I explain what diabetes is. I tell them they might feel tingling in their hands and feet, or have a dry mouth and dry nose. So, I counsel them to go get a checkup*." (Ward No.6, Phase 2)

Mixed-methods integration suggests that FCHV's increased confidence in delivering diabetes self-management support was not only from gaining knowledge but also from their identity as a health educator in the community. The training fueled their validation of lived experience and formalized their role, leading to greater trust and impact in the community.

Theme 4: Training and education

In phase 1, many FCHVs identified lack of training and education as a key barrier to providing self-management support within their communities. By phase 2, FCHVs were more confident and capable in their roles which suggest that the training not only improved their diabetes related knowledge but also strengthened their ability to implement that knowledge in the real world setting.

In qualitative findings, participants reflected on their past experiences, noting a lack of structured or follow-up training and expressing the need for a more committed and systematic approach to skill-building. One participant shared:

> Quote 8: "*Some organizations come and provide training just once, then never return. As a result, we do not get updated*." (Ward No. 6, Phase 1)

This sentiment was reflected in several participants' responses where they pointed a gap between initial and ongoing support to build FCHVs' capacity. One participant emphasized that without regular education, FCHVs are left unprepared to support others, underlining the critical link between training and competency.

> Quote 9: "*We can become capable if we receive proper training. Without knowledge, how can we offer suggestions and advice to others?"* (Ward No. 3, Phase 1)

By Phase 2, FCHVs expressed deep appreciation for the training. Many found it helpful and shared how they felt empowered to apply their knowledge. The training not only improved their technical understanding but also sparked curiosity and a stronger sense of responsibility toward implementing the learnings within their communities. One participant articulated the importance of comprehensive training as a foundation for confidence and outreach.

> Quote 10: "*We can only spread knowledge in the community if we receive complete training. First, we need to feel fully confident and say to ourselves, 'Yes, I can go and work in the community*." (Ward No. 3, Phase 2)

The integration of quantitative and qualitative findings indicates that training served not only to address immediate knowledge deficits but also to cultivate a deeper commitment among FCHVs in their role as community health advocates. Their growing call for ongoing education highlights a shift from viewing training as a one-time intervention to recognizing it as a necessary foundation for sustained capacity and credibility in their community-facing roles.

Theme 5: Barriers and motivators for community-based self-management support

In phase 1, some FCHVs identified community's belief and attitude, engagement in multiple programs and lack of incentive and motivation as barriers to provide self-management support. The barriers persisted in Phase 2 and perception of inadequate incentives worsened (Table 2).

Qualitative analysis revealed three subthemes under the main theme barriers and motivators for community-based self-management support: Workload, inadequate incentives and community's receptiveness towards counseling.

Subtheme 1: Workload

In Phase 1, FCHVs highlighted the sheer volume of responsibilities they managed, which was difficult to manage. Many described being pulled in multiple projects—supporting maternal health, child immunization, elder care, education outreach, which made it difficult to retain new information or dedicate focused time to lifestyle counseling. One participant shared how juggling dozens of tasks diluted their capacity to absorb and apply training content:

> Quote 11: "*There is a need for regular training. A one-time training is not enough. It's difficult to retain all the information from a single session over the years. We need training every six months. Since we are involved in so many different tasks, we tend to forget. As Female Community Health Volunteers, we are responsible for around 35 different types of work—ranging from caring for pregnant women to supporting the elderly.*" (Ward no. 6, Phase 1)

Although this subtheme of overwhelming work demand was prominent in Phase 1 discussions, it notably did not emerge in Phase 2, suggesting that either the training or the study's focus may have shifted FCHVs' perceptions of workload.

Subtheme 2: Inadequate incentives

Nearly all FCHVs in Phase 1 raised concerns about the lack of adequate incentives tied to their volunteer work. While they were deeply committed to serving their communities, they felt that modest financial or material support would bolster their motivation and help sustain their engagement over time.

Quote 12*: "We need training, we have to work day and night, so we should have services and facilities accordingly, and there should be remuneration." (Ward no. 3, Phase 1)*

Quote 13: "*There should be provision of training along with increased incentives though there is no salary for us. Rs 400(~$3) is not sufficient for us, at least there should be provision of Rs. 1000(~$7.10) so we will be encouraged to do our responsibility*." (Ward No.6, Phase 1)

Subtheme 3: Community's receptiveness towards counseling

Both Phase 1 and Phase 2 discussions shed light on how community attitudes shaped FCHVs' ability to counsel effectively, but the tenor shifted markedly over time.

In phase 1, FCHVs expressed frustration about lack of authority figures like physicians in the field with them, which could lead to community members being unreceptive to FCHVs counseling expertise, making it challenging for the FCHVs to establish their credibility and deliver impactful counseling.

Quote 14: "*Community people said that we only do counseling without providing anything. So, it would be better if there is provision of medicine*." (Ward No. 6, Phase 1)

By Phase 2, however, FCHVs described a far more receptive audience. They observed that residents were not only listening to their recommendations but also implementing them. One

FCHV in Phase 2 highlighted the tangible feedback they received—community members affirming that "*the things we were saying was good*"—which reinforced the value of their counseling.

> Quote 15: "*Some say that they are doing checkup as per our guidance, some said they are fine, some said their sugar level is increasing, and doctor is suggesting having control on diet. They said that the things we were saying was good*." (Ward No. 4, Phase 2)

Another participant articulated the growing receptiveness of community members towards FCHVs' self-management support:

> Quote 16: "*They follow our suggestions and advices, they listen to us, they said we are right and they do checkup*." (Ward No 4, Phase 2)

The integration of qualitative and quantitative findings reveals that while systemic barriers such as workload and inadequate incentives persisted, the increasing receptiveness of the community emerged as a powerful motivator that helped sustain FCHVs' engagement in self-management support.

Discussion

This mixed method study evaluated FCHV's knowledge, perceived self-efficacy, and barriers to providing diabetes self-management support in rural Nepal before and after structured training program. Guided by the HBM, we found that even a brief, skills-based training enhanced FCHVs' understanding of diabetes and its risk factors and increased their confidence in supporting community members. Although persistent barriers such as limited incentives were identified, the training also contributed to community receptiveness towards FCHVs as diabetes-

related supporters. Our findings suggest that FCHVs are both willing and able to expand their role to include diabetes care, and that short, targeted training sessions coupled with ongoing refresher training and appropriate incentives represents a feasible strategy in NCDs management in resource-constrained settings.

A growing body of evidence supports community-based management of NCDs, especially in LMIC where there is a critical shortage of human resources [4, 12, 14, 15, 21]. A study showed that CHWs can partially fill the void from severe human resources shortage as they bridge between community and healthcare system [22]. Their role in behavior change interventions has demonstrated clinical effectiveness in multiple NCDs [4, 12, 14]. A randomized trial in Nepal showed significant improvements in glycemic control among individuals with diabetes who received CHW-led interventions [4]. Similarly, trials conducted in other LMICs, such as India and South Africa, have also reported that CHW-delivered programs led to improved glucose levels, blood pressure control, and adherence to treatment regimens in individuals with diabetes and other NCDs [21, 23, 24]. These findings highlight the potential of CHWs to not only provide self-management support but also drive measurable clinical outcomes in resource constrained settings. In this context, evaluating the readiness of CHW to deliver the service is important and timely, especially given the heterogeneity of their skills and background. The current study contributes that even a short and targeted program can significantly boost CHWs' knowledge and confidence, especially if coupled with repeat training.

FCHVs are integral members of their communities, providing various services that extend beyond a single health condition. They live in the same communities they serve and sometimes their service extends to neighboring areas. Despite establishing themselves as respected members of the community, FCHVs face challenges in a patriarchal society [11]. Our qualitative analysis

highlighted that after the training, FCHVs perceived the community to be more receptive towards their counseling. This further motivated FCHVs to continue learning and providing care in their communities.

In our study, we noted barriers such as limited expertise, inadequate incentives, and heavy workloads persisted. FCHVs themselves emphasized the need for more comprehensive and frequent training to enhance their skills and credibility. Similarly, FCHVs said that the incentives as per local norms were not adequate. These barriers mirror findings from other community health worker programs including Nepal and similar settings, where lack of incentives and structural support have been shown to limit program effectiveness [25, 26]. Addressing these constraints through system-level strategies—such as appropriate incentive program, recognition within the health system, and provision of job benefits or counseling materials, will be essential for maintaining motivation and ensuring sustainability. At the same time, increasing receptivity of community members toward FCHVs underscores their unique potential to deliver diabetes self-management effectively if adequate support mechanisms are in place.

The findings from this study have important implications for policy and practice in Nepal and other resource-limited settings. Integrating diabetes education and counseling into the existing scope of FCHVs is feasible if training, incentives and workload and other systemic support are tailored to FCHVs needs and capacity. If substantiated, structured and targeted training modules, such as the one evaluated in this study, could be incorporated into the national FCHV training curriculum and reinforced through periodic refresher sessions.

This study has several strengths. We conducted training and evaluations in two phases. The second phase was after FCHVs had an opportunity to use the skills they learned at Phase 1. By

employing a concurrent qualitative dominant embedded study design, we were able to gain depth and context through qualitative insights, while also incorporating quantitative data. At the same time, the study has several limitations. First, the training primarily emphasized knowledge rather than skills building, with limited opportunity for structured assessments of counseling fidelity, largely due to resource constraints. Although concepts such as motivational interviewing and shared decision-making were introduced, the training was not designed to provide a deeper understanding or supervised practice of these methods. Second, the COVID-19 pandemic created challenges for community engagement, contributing to variability in FCHVs' opportunities to apply their training between phases. Third, the quantitative evaluation was underpowered for inferential testing, so the results should be interpreted as descriptive trends rather than definitive effects that added to the detailed qualitative evaluation. Finally, as with many self-report surveys, there may have been risks of social desirability bias. Despite these limitations, this study provides important insights into the potential for brief, repetitive, skills-oriented trainings for FCHVs in rural Nepal.

Conclusions

Our findings highlighted the value of focused and repetitive training in FCHVs' role in the prevention and management of diabetes in resource limited settings. However, sustainability and impact of FCHVs led interventions are shaped by the structural and social conditions such as incentives, workload and the receptiveness of the community in FCHVs' capacity in diabetes care. As burden of diabetes and other NCDs continue to rise, strengthening interventions led by FCHVs that are socially and structurally supported, offer a way forward for more sustainable and equitable care.

References

1. Organization WH. Assessing national capacity for the prevention and control of noncommunicable diseases: report on 2023 global survey. Geneva: World Health Organization; 2025.
2. Federation ID. IDF Diabetes Atlas. Brussels, Belgium: International Diabetes Federation; 2025.
3. Karachaliou F, Simatos G, Simatou A. The Challenges in the Development of Diabetes Prevention and Care Models in Low-Income Settings. Front Endocrinol (Lausanne). 2020;11:518.
4. Gyawali B, Sharma R, Mishra SR, Neupane D, Vaidya A, Sandbæk A, et al. Effectiveness of a Female Community Health Volunteer-Delivered Intervention in Reducing Blood Glucose Among Adults With Type 2 Diabetes: An Open-Label, Cluster Randomized Clinical Trial. JAMA Netw Open. 2021;4(2):e2035799.
5. Prevention CfDCa. Division for Heart Disease and Stroke Prevention's Best Practices Clearinghouse. Division for Heart Disease and Stroke Prevention; 2022, February 28.
6. Wu Y, Ding Y, Tanaka Y, Zhang W. Risk factors contributing to type 2 diabetes and recent advances in the treatment and prevention. Int J Med Sci. 2014;11(11):1185-200.
7. Schaaf M, Warthin C, Freedman L, Topp SM. The community health worker as service extender, cultural broker and social change agent: a critical interpretive synthesis of roles, intent and accountability. BMJ Glob Health. 2020;5(6).
8. Manandhar S, Adhikari RP, Acharya A, Pollifrone MM, Nepali LB, Darji P, et al. Health Mothers' Groups in Nepal: Barriers, Facilitators, and Recommendations. Curr Dev Nutr. 2022;6(5):nzac039.
9. Kandel N, Lamichhane J. Female health volunteers of Nepal: the backbone of health care. Lancet. 2019;393(10171):e19-e20.
10. Alam K, Tasneem S, Oliveras E. Performance of female volunteer community health workers in Dhaka urban slums. Soc Sci Med. 2012;75(3):511-5.
11. Khatri RB, Mishra SR, Khanal V. Female Community Health Volunteers in Community-Based Health Programs of Nepal: Future Perspective. Front Public Health. 2017;5:181.
12. Adhikari TB, Gyawali B, Rijal A, Sapkota A, Högman M, Karki A, et al. Community-based management of chronic obstructive pulmonary disease in Nepal-Designing and implementing a training program for Female Community Health Volunteers. PLOS Glob Public Health. 2022;2(3):e0000253.
13. Gyawali B, Mishra SR, Neupane D, Vaidya A, Sandbæk A, Kallestrup P. Diabetes management training for female community health volunteers in Western Nepal: an implementation experience. BMC Public Health. 2018;18(1):641.
14. Neupane D, McLachlan CS, Mishra SR, Olsen MH, Perry HB, Karki A, et al. Effectiveness of a lifestyle intervention led by female community health volunteers versus usual care in blood pressure reduction (COBIN): an open-label, cluster-randomised trial. Lancet Glob Health. 2018;6(1):e66-e73.
15. Rawal LB, Sun Y, Dahal PK, Baral SC, Khanal S, Arjyal A, et al. Engaging Female Community Health Volunteers (FCHVs) for cardiovascular diseases risk screening in Nepal. PLoS One. 2022;17(1):e0261518.
16. Neupane G, Acharya S, Bhattarai M, Upadhyay A, Belbase B, Bhandari M, et al. Study, Design, and Rationale of Noncommunicable Diseases in Nepal (NCD Nepal) Study: A Community-Based Prospective Epidemiological and Implementation Study in Rural Nepal. Glob Adv Health Med. 2020;9:2164956120917379.
17. Janz NK, Becker MH. The Health Belief Model: a decade later. Health Educ Q. 1984;11(1):1-47.
18. Jones CL, Jensen JD, Scherr CL, Brown NR, Christy K, Weaver J. The Health Belief Model as an explanatory framework in communication research: exploring parallel, serial, and moderated mediation. Health Commun. 2015;30(6):566-76.
19. Fereday J, Muir-Cochrane E. Demonstrating Rigor Using Thematic Analysis: A Hybrid Approach of Inductive and Deductive Coding and Theme Development. 2006;5(1):80-92.

20. Schoonenboom J, Johnson RB. How to Construct a Mixed Methods Research Design. Kolner Z Soz Sozpsychol. 2017;69(Suppl 2):107-31.
21. Jeet G, Thakur JS, Prinja S, Singh M. Community health workers for non-communicable diseases prevention and control in developing countries: Evidence and implications. PLoS One. 2017;12(7):e0180640.
22. Abrahams-Gessel S, Denman CA, Montano CM, Gaziano TA, Levitt N, Rivera-Andrade A, et al. Training and supervision of community health workers conducting population-based, noninvasive screening for CVD in LMIC: implications for scaling up. Glob Heart. 2015;10(1):39-44.
23. Basu P, Mahajan M, Patira N, Prasad S, Mogri S, Muwonge R, et al. A pilot study to evaluate home-based screening for the common non-communicable diseases by a dedicated cadre of community health workers in a rural setting in India. BMC Public Health. 2019;19(1):14.
24. Jarvis JD, Kataria I, Murgor M, Mbau L. Community Health Workers: An Underappreciated Asset to Tackle NCD. Glob Heart. 2016;11(4):455-7.
25. Maru S, Nirola I, Thapa A, Thapa P, Kunwar L, Wu WJ, et al. An integrated community health worker intervention in rural Nepal: a type 2 hybrid effectiveness-implementation study protocol. Implement Sci. 2018;13(1):53.
26. Singh D, Negin J, Otim M, Orach CG, Cumming R. The effect of payment and incentives on motivation and focus of community health workers: five case studies from low- and middle-income countries. Hum Resour Health. 2015;13:58.

Supporting Information

S1_Quantitative Survey

S2_Qualitative Questionnaire

S3_theme-suthemes and quotes

S4_Translated Quotes

Study Timeline

Click here to access/download;Figure;Fig 1.tif

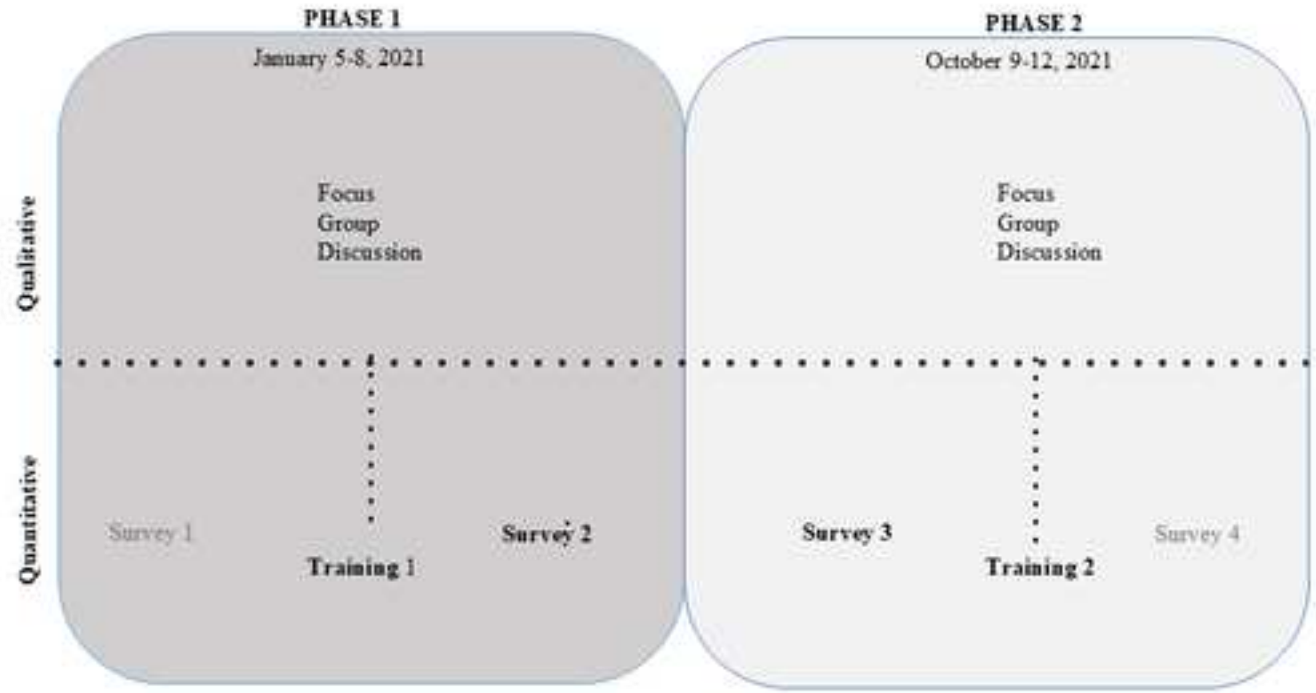

Flowchart of mixed method design

Click here to access/download;Figure;Fig 2.tif

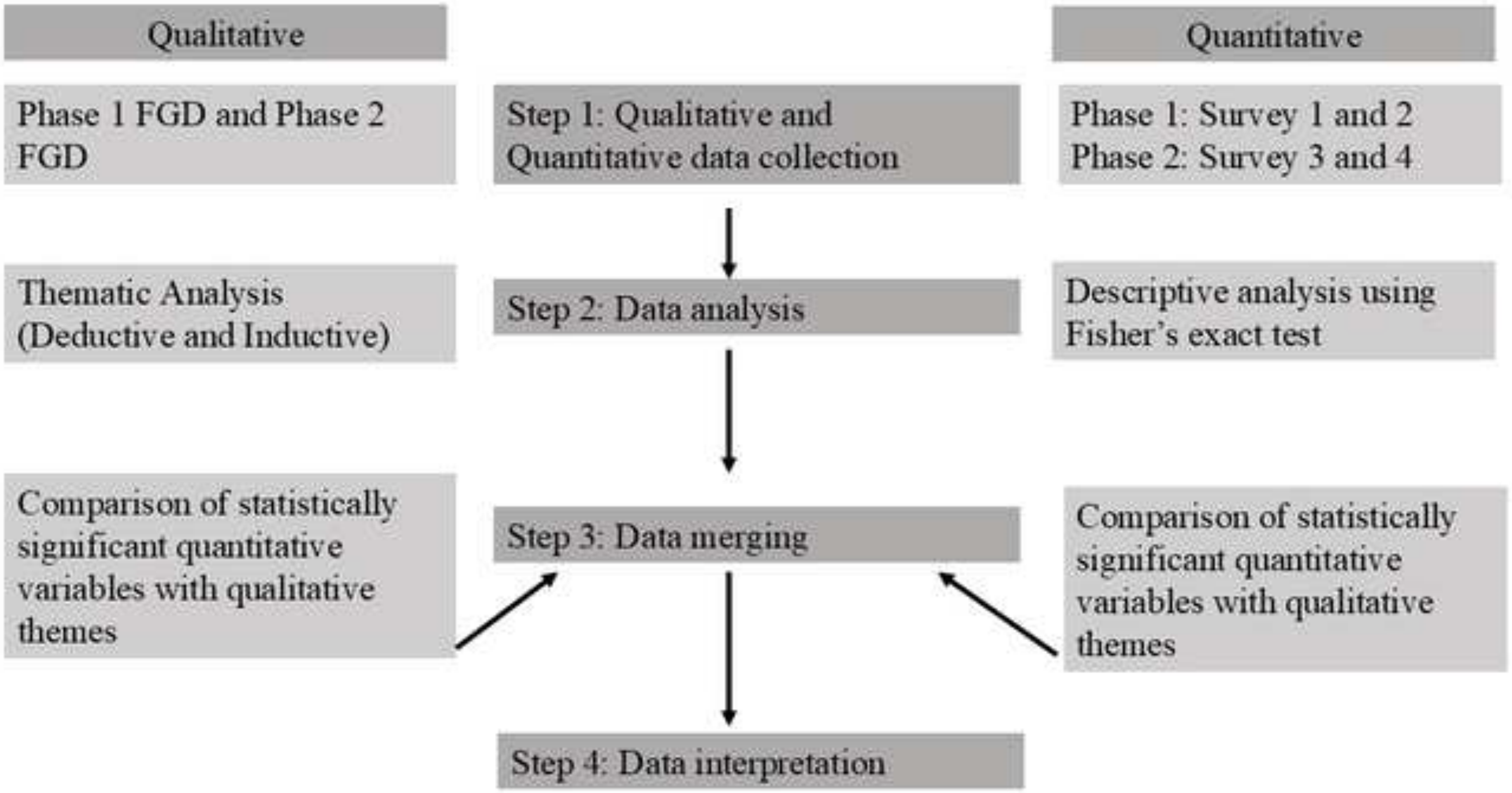

Click here to access/download
Supporting Information
S3_Theme-subtheme and quotes.xlsx

Qualitative Question

Click here to access/download
Supporting Information
S2 .Qualitative_FGD question.docx

Click here to access/download
Supporting Information
S 4. Translated Quotes.docx

Quantitative Questions

Click here to access/download

Supporting Information

S1. Quantitative survey questions.docx
