# Supplementary material for: Female community health volunteers’ knowledge and confidence in providing community-based diabetes self-management support in Nepal: A biphasic mixed method evaluation
Source: PLOS Glob Public Health. 2026 Mar 12;6(3):e0006089. doi: 10.1371/journal.pgph.0006089 (PMC12981428; doi:10.1371/journal.pgph.0006089)
Supplement: Attachment — Submitted filename: Response to Reviewers.docx [file pgph.0006089.s007.docx]

**Reviewer’s comments**

**Reviewer #1**

**Major Concerns:**

**Comment #1 The sample is small (n=28 from one district, four wards). Selection was convenience-based due to COVID-19 rules. This limits representativeness.**

**Response**: We greatly appreciate the reviewer’s comments. The reviewer raised important **questions about sample size and COVID-19 impact**. In qualitative analysis of focus group discussion (FGD), saturation can be achieved with as low as four focus groups [Qual Health Res. 2019 Jan 10;29(10):1483–1496]. We had 28 participants in a total of eight focus groups across two phases. Please find below relevant text in the limitation section of the manuscript.

Lines 439-444 (Discussion, last paragraph)

“We achieved thematic saturation during qualitative analysis. At the same time, the study has several limitations. First, COVID restrictions affected our recruitment, and thus, the sampling was not truly purposeful, which is preferred in qualitative work. However, participating FCHVs had a range of age, education and experience that still allowed us to rigorously evaluate our research questions.”

**Broad claims about FCHVs in Nepal need toning down. Frame the work clearly as exploratory or pilot.**

**Response**: We are very appreciative of the opportunity to respond to the reviewer’s **comments about study conclusions**. We agree with toning down the conclusions. However, we respectfully decline this work as pilot nature. Please see our response about sample size and recruitment above. We were able to rigorously evaluate our study questions in our recruited participants and achieved thematic saturation. In qualitative studies, our goal is to understand underlying phenomenon. The goal is not to generalize the findings, but a related concept is transferability of findings. Transferability is left for readers to decide if the findings are applicable in their contexts [*Research on Social Work Practice*, *35*(1), 102-110. <https://doi.org/10.1177/10497315241256560>]. We agree with the reviewer that given the possible heterogeneity of FCHVs in other places in Nepal, similar studies elsewhere would be welcome. Please find relevant text in the manuscript as follows, with underlined words showing the important change.

Lines 55-59 (Abstract)

“These findings suggest that FCHVs may be willing to take a greater role in diabetes self-management support, and that even short training sessions have the potential to enhance their confidence and knowledge, particularly if our findings are further substantiated.”

Lines 386-388 (Discussion, first paragraph)

“This mixed method study evaluated FCHV’s knowledge, perceived self-efficacy, and barriers to providing diabetes self-management support in rural Western Nepal before and after structured training program”

Additional relevant text in lines 392-395 (Discussion, first paragraph)

“Our findings suggest that FCHVs are both willing and able to expand their role to include diabetes care, and that short, targeted training sessions coupled with ongoing refresher training and appropriate incentives represent a feasible strategy in NCDs management in resource-constrained settings, especially if our findings are further substantiated.”

Lines 429-432 (Discussion)

“The findings from this study have important implications for policy and practice in Nepal and other resource-limited settings, if our findings are further substantiated. Integrating diabetes education and counseling into the existing scope of FCHVs can be feasible if training, incentives and workload and other systemic support are tailored to FCHVs needs and capacity.”

Lines 444-446 (added text in the Discussion, last paragraph)

“Furthermore, our evaluation was limited to one district. We encourage additional studies to evaluate transferability of our findings to other places in Nepal, especially given the potential heterogeneity of FCHVs knowledge, skills and education.”

Lines 462-469 (Conclusions, manuscript)

Our findings highlighted the potential of focused and repetitive training in FCHVs’ role in the prevention and management of diabetes in resource limited settings”…“Transferability of these findings to other areas of Nepal should be evaluated. As burden of diabetes and other NCDs continue to rise, strengthening interventions led by FCHVs that are socially and structurally supported can offer a way forward for more sustainable and equitable care.

**Comment #2 The study occurred during COVID-19 restrictions. This affected recruitment, group size, and possibly outcomes. The current brief mention is not enough. Expand the limitations section with specific pandemic effects.**

**Response:** Thank you for your comment. We have expanded our limitation section according to your suggestions.

Lines 441-446

“First, COVID restrictions affected our recruitment, and thus, the sampling was not truly purposeful, which is preferred in qualitative work. However, participating FCHVs had a range of age, education and experience that still allowed us to rigorously evaluate our research questions. Furthermore, our evaluation was limited to one district. We encourage additional studies to evaluate transferability of our findings to other places in Nepal, especially given the potential heterogeneity of FCHVs knowledge, skills and education. Second, the training primarily emphasized knowledge rather than skills building, with limited opportunity for structured assessments of counseling fidelity, largely due to resource constraints. Although concepts such as motivational interviewing and shared decision-making were introduced, the training was not designed to provide a deeper understanding or supervised practice of these methods. Third, the COVID-19 pandemic created challenges for community engagement, contributing to variability in FCHVs’ opportunities to apply their training between phases.”

**Comment #3 No control group exists.**

**Response**: Thank you for your comment. Our study is evaluating an implementation question about an evidence-based intervention as prior randomized trial has shown that FCHVs led self-management support improves glycemic control [JAMA Netw Open. 2021;4(2):e2035799]. Link: <https://jamanetwork.com/journals/jamanetworkopen/fullarticle/2775660> Having an additional control arm in our study for quantitative evaluation of the impact of training on FCHVs in both phases would have helped us understand the issue of temporal trends and regression towards the mean. However, given limited resources and particularly the presence of qualitative evaluation that provides richer details, we did not see the justification of having the control arm.

**Phase 2 results combine initial training, field experience, and refresher effects. This makes it hard to isolate the impact of one short training.**

**Response**: We appreciate the comment. Regarding understanding the effect of isolated initial training at phase 1, we can look at the results between Survey 1 (S1) and Survey 2 (S2) in Table 2. S1 occurred prior to the initial training and S2 occurred immediately after the initial training. As we can see, there were statistically significant improvements in all questions that we evaluated during S1 and S2. Nevertheless, we warn caution in interpreting these quantitative data given small sample size in this qualitative dominant mixed method study. Similarly, we can also look at the ‘decay’ in knowledge over nine months, when comparing S2 to S3 that occurred immediately before the refresher training.

Relevant text in lines 454-456 in the last paragraph of the Discussion section

“Fifth, the quantitative evaluation was underpowered for inferential testing, so the results should be interpreted as descriptive trends rather than definitive effects that added to the detailed qualitative evaluation.”

**Reviewer #2**

**Congratulations on the success of your intervention—this is a strong and well-written article. I have a few minor questions and suggestions that may be helpful to address prior to publication:**
**Comment # 1. You note an 11-month interval between Phases I and II in both the abstract (line 44) and the Methods section (line 157); however, the period from January to October appears to be nine months. Please clarify this discrepancy in the timeline. Additionally, the mention of date in the abstract may not be necessary. If you decide to keep it, please also include the dates for Phase II.**

**Response**: We appreciate the reviewer picking up this important oversight on our part, which we have corrected now to nine months throughout the manuscript. Additionally, we also mentioned the time difference and removed the date from the abstract.

**Comment # 2**. **The introduction effectively frames diabetes as a global concern and highlights the role of community health workers as a bridge between communities and the health system. It would strengthen the paper to include additional context on the burden of diabetes in Nepal—particularly in the study region—and the unique causes and risk factors relevant to this setting.**

**Response:** Thank you for this important comment. We did not find any published study showing diabetes burden in our study region, Dang, Nepal. However, based on our own preliminary unpublished data, we found diabetes prevalence of 8.4% among 2867 adults in Dang using capillary blood glucose, which is similar to national data. However, we believe that the actual prevalence will be higher once we analyze our data using all other possible definitions of diabetes (hemoglobin A1c level, those taking medications etc). We have provided additional information about diabetes burden in Nepal in the revised introduction section, but not about the local study region, given the preliminary nature of our unpublished data.

Lines 62-73

“Non communicable diseases (NCDs) such as heart disease, stroke, and diabetes are the leading causes of mortality and morbidity globally. Approximately 106.9 million adults aged 20- 79 years are living with diabetes in South-East Asia. This number is projected to increase by 73% reaching 185 million by 2050 . Nepal, a small developing country in South- Asia, with a population of 26 million has the fourth highest number of adults aged 20 -79 years with diabetes. The number of adults with diabetes in Nepal is predicted to increase from 1.3 million to 2.4 million by 2050 . Although the burden is increasing rapidly, there are limited resources and healthcare capacities to meet the growing demand for diabetes care. Managing diabetes in these settings presents numerous challenges, including limited healthcare professionals, medications, and challenges in improving patients’ self-management skills. Given these challenges, community-based, sustainable and scalable diabetes care approaches that do not solely rely on traditional healthcare providers can augment diabetes care.”

**Comment # 3**. **While the study context is described, the specific purpose or objective of the paper is not stated explicitly. Clarifying this in the introduction would help orient the reader.**

**Response**: Thank you for your comment. We have now clarified the specific purpose in the introduction section.

Lines 91-100

“Recently there has been growing interest in expanding their involvement to non-communicable diseases (NCDs), including diabetes self-management support. However, before expanding such programs, evaluating whether FCHVs possess the necessary knowledge and confidence in delivering diabetes care is essential. An assessment of their training needs, potential barriers, and ability to retain information is important to understand the program’s success. This is particularly relevant given the considerable variability in FCHV’s education and background. Therefore, we conducted a biphasic concurrent, embedded mixed-methods evaluation to understand FCHVs’ knowledge, perceived self-efficacy and barriers in providing diabetes self-management support and to evaluate the impact of a short training on change in knowledge at various time points.”

**Comment # 4**. **I am interested in the 9-item survey assessing diabetes knowledge and confidence (line 140). Was this a newly developed instrument or an adaptation of an existing, validated diabetes knowledge questionnaire? If self-developed, could you briefly describe the steps taken to assess its validity?**

**Response**: Thank you for this important comment. The survey was newly developed. Our goal was to use it as a simple tool to evaluate relevant constructs for our study. We have added the following explanation in the method section.

Lines 137-149.

“The HBM proposes six socio-behavioral constructs influencing health behaviors. We selected three constructs – knowledge, self-efficacy, and barriers - to understand how FCHVs perceive and provide self-management support reflecting internal and external factors that influence their practices. The survey had five items for knowledge, three items for self-efficacy and one item for barriers. For each phase, we included only relevant items. Our working knowledge of FCHVs informed us in selecting relevant survey questions and responses. We first created the survey in English. SB and SS independently translated them into Nepali language, that was reviewed and reconciled by the larger investigative team. Subsequently, we pilot tested the survey among study staff and refined for clarity and relevance. Then we pre-tested the survey among a few lay individuals with similar health literacy and educational backgrounds as the FCHVs from the study area. This resulted in minor additional refinement for clarity and flow. We used similar process for the interview guides.”

We also added the following in the limitation section of the manuscript.

Lines 456-457

“Furthermore, although we used a rigorous process to develop the survey, we did not conduct formal psychometric evaluation.”

**Comment # 5**. **The study design is described as a “concurrent, embedded mixed-methods” approach in the abstract (line 41) and Methods section (line 186), but as “concurrent” in the introduction (line 93). Please ensure consistency in terminology throughout the manuscript.**

**Response**: Thank you for the thorough review. We have now made the changes according to your suggestions.

**Comment # 6. The formatting of the brackets in the final column of Table 1 appears inconsistent (this may be a display issue on my end). Please review this and consider standardizing the table design for consistency.**

**Response**: Thank you. The word file shows correct formatting of brackets, but we noted that the pdf file generated by the publisher showed inconsistency. We will work with the publisher on this, if this continues to be an issue.

**Comment # 7. I hope these comments are helpful, and I look forward to seeing the revised version.**

**Response**: We appreciate the reviewer’s comments which have improved the quality of our manuscript.
